# Property Analysis of the Real-Time Uncalibrated Phase Delay Product Generated by Regional Reference Stations and Its Influence on Precise Point Positioning Ambiguity Resolution

**DOI:** 10.3390/s17051162

**Published:** 2017-05-19

**Authors:** Yong Zhang, Qing Wang, Xinyuan Jiang

**Affiliations:** 1 School of Instrument Science and Engineering, Southeast University, Nanjing 210096, China; zhangyong136l@163.com; 2 Germany Research Center For Geosciences (GFZ), Telgrafenberg, 14473 Potsdam, Germany; jxinyuan@gfz-potsdam.de

**Keywords:** real-time precise point positioning (RTPPP), real-time precise point positioning ambiguity resolution (RTPPP-AR), uncalibrated phase delay (UPD), regional station

## Abstract

The real-time estimation of the wide-lane and narrow-lane Uncalibrated Phase Delay (UPD) of satellites is realized by real-time data received from regional reference station networks; The properties of the real-time UPD product and its influence on real-time precise point positioning ambiguity resolution (RTPPP-AR) are experimentally analyzed according to real-time data obtained from the regional Continuously Operating Reference Stations (CORS) network located in Tianjin, Shanghai, Hong Kong, etc. The results show that the real-time wide-lane and narrow-lane UPD products differ significantly from each other in time-domain characteristics; the wide-lane UPDs have daily stability, with a change rate of less than 0.1 cycle/day, while the narrow-lane UPDs have short-term stability, with significant change in one day. The UPD products generated by different regional networks have obvious spatial characteristics, thus significantly influencing RTPPP-AR: the adoption of real-time UPD products employing the sparse stations in the regional network for estimation is favorable for improving the regional RTPPP-AR up to 99%; the real-time UPD products of different regional networks slightly influence PPP-AR positioning accuracy. After ambiguities are successfully fixed, the real-time dynamic RTPPP-AR positioning accuracy is better than 3 cm in the plane and 8 cm in the upward direction.

## 1. Introduction

With the continuous development of Global Navigation Satellite System (GNSS) positioning technology, there are more and more methods for obtaining high-accuracy position information in a real-time manner [1]. Specifically, Precise Point Positioning (PPP) technology can realize global high-accuracy positioning using a single site and has been widely used in many areas [2,3,4,5,6,7,8]. Bismuth el al. [6,7,8,9,10,11], have applied PPP to determine satellite orbits. Chen et al. [12] used the PPP for Sea level monitoring. Banville and Langley [13] applied an instantaneous cycle-slip correction method for Real-Time Precise Point Positioning (RTPPP). Li et al. [14] presented a GPS + GLONASS + BeiDou + Galileo four-system model to fully exploit the observations of all four navigation of these satellite systems for RTPPP.

These years, Precise Point Positioning Ambiguity Resolution (PPP-AR) with higher accuracy has been greatly developed [9,15,16,17,18]. PPP-AR can shorten the convergence time based on the fixed ambiguity information and obtain cm-level real-time high-accuracy absolute coordinates in a short time, thus having wider application prospects compared with float PPP. Due to the precise point positioning mode, satellite UPDs have made PPP precise ambiguities lose their integer property [15,16,17,18,19,20]. Ge et al. [16] successfully realized PPP-AR using single-difference data between satellites with daily observations. Bertiger et al. [9] submitted undifferenced ambiguities which contain Uncalibrated Phase Delay (UPD) to PPP users for ambiguity resolution. Laurichesse et al. [18] and Collins et al. [17] have provided a satellite clock product which could recover the integer property of undifferenced ambiguities, but not all satellite clock products have integer properties. Geng et al. [7] proved that these above methods are equivalent to each other.

Ge et al. [16] obtained the UPDs from a global reference network and applied the UPDs to PPPAR. The precondition for obtaining a PPP-AR solution is the UPD estimation [16], and real-time UPD estimation for RT-PPPAR. The UPD estimation stations have physical distribution as well as quantity properties. Although many researchers have studied the properties of UPD in recent years [21,22], most of these studies are based on post-processing mode. For implementing Real-time Precise Point Positioning Ambiguity Resolution (RTPPP-AR), it is necessary to correct the real-time UPD to obtain the precise ambiguity to recover the integer characteristics of the precise ambiguities [19,20,21,22,23].

In this paper, we will focus on the properties of real-time UPD estimated by regional stations. The precise ambiguity integer property will be recovered by the real-time UPD product for RTPPP-AR mode, and the performance of RTPPP-AR positioning will be analyzed in details for the real-time UPD products estimated by different regional stations. The paper is organized as follows: Section 2.1 describes the theory of how to fix the ambiguity based on UPD products. Section 2.2 presents the methods for wide-lane and narrow-lane UPD estimation, respectively. Section 3 details the properties of the real-time UPD product and its influence on real-time PPP ambiguity resolution based on real-time experiments and Section 4 draws the conclusions.

## 2. RTPPP-AR and RT UPD Estimation

### 2.1. RTPPP-AR Method

The UPD between the satellite terminal and the receiver terminal cannot be separated from the ambiguity parameters, thus making ambiguities $N_{r,i}^{s}$ in the precise phase observation value become float solution, as shown in the following formula [16,24,25,26]:

(1) $$B_{r,i}^{s}=N_{r,i}^{s}+b_{r,i}-b_{r,i}^{s}$$

where the subscripts *r* and *s* represent the receiver and the satellite, respectively, i = (1,2,3) is the frequency signal, $N_{r,i}^{s}$ is the ambiguity, $b_{r,i}$ is the UPD of the receiver at frequency i, $b_{r,i}^{s}$ is the UPD of the satellite at frequency i, and $B_{r,i}^{s}$ is actual floating-pointing solution ambiguity.

In RTPPP-AR method, the following two modes are usually adopted for uncalibrated phase delay $b_{r,i}$ of the receiver: (1) the uncalibrated phase delay is coupled into the clock correction of the receiver positioning; (2) uncalibrated phase delay $b_{r,i}$ of the receiver is eliminated by the single-difference between two satellites. What’s more, the single-difference between two satellites method can not only eliminate the uncalibrated phase delay of the receiver, but also form the single-difference ambiguity, and the single-difference ambiguities, which can eliminate certain residual error influence, have higher search efficiency in ambiguity resolution.

In PPP, the ionosphere free combined model is usually adopted to eliminate the ionosphere influence for positioning [27,28]. Due to the combination coefficient, ambiguity $N_{r,IF}^{s}$ of the ionosphere free combined model is not an integer. In actual RTPPP-AR process, $N_{r,IF}^{s}$ is usually decomposed into wide-lane $N_{r,WL}^{s}$ and narrow-lane $N_{r,1}^{s}$ ambiguity (the ambiguity coefficient is narrow lane, so $L_{1}$ ambiguity is treated as narrow-lane ambiguity) for fixing [29,30,31,32]; they have the integer characteristic, and LAMBDA [33] can be used to search the narrow-lane ambiguities. Compared with short-baseline relative positioning, more parameters need to be estimated in PPP and some of the parameters such as ambiguities, receiver clock and zenith tropospheric delay are highly correlated. It is more difficult to fix all ambiguity parameters reliably for PPP than in DD-based relative positioning [30], so a partial ambiguity fixing strategy [30,31] has been used to first fix optimum ambiguities cell in order to shorten the ambiguity fixing time and improve the accuracy and reliability of ambiguity solutions in PPP-AR.

Then ambiguity $N_{r,IF}^{s}$ of the ionosphere free combined model is recovered after $N_{r,WL}^{s}$ and $N_{r,1}^{s}$ have been fixed:

(2) $$\begin{array}{l} \lambda_{IF}\cdot N_{r,IF}^{s}=\frac{cf_{2}}{f_{1}^{2}-f_{2}^{2}}N_{r,WL}^{s}+\frac{c}{f_{1}+f_{2}}N_{r,1}^{s} \end{array}$$

where $N_{r,WL}^{s}=N_{r,1}^{s}-N_{r,2}^{s}$, $\lambda_{IF}=\frac{c}{f_{1}^{2}-f_{2}^{2}}$; considering the UPD, Equation (2) is converted as follows:

(3) $$\lambda_{IF}\cdot {\tilde{N}}_{r,IF}^{s}=\frac{cf_{2}}{f_{1}^{2}-f_{2}^{2}}(N_{r,WL}^{s}+b_{r,wl}-b_{r,wl}^{s})+\frac{c}{f_{1}+f_{2}}(N_{r,1}^{s}+b_{r,1}-b_{r,1}^{s})$$

Equation (3) is converted into single-difference by two satellites mode [16,24] as follows:

(4) $$\lambda_{IF}\cdot \nabla {\tilde{N}}_{r,IF}^{j,k}=\frac{cf_{2}}{f_{1}^{2}-f_{2}^{2}}(\nabla N_{r,WL}^{j,k}-\nabla b_{wl}^{j,k})+\frac{c}{f_{1}+f_{2}}(\nabla N_{r,1}^{j,k}-\nabla b_{1}^{j,k})$$

Only when single-different WL ambiguities $\nabla {\overset{⌢}{n}}_{WL}^{j,k}$ and single-different NL ambiguities $\nabla {\overset{⌢}{n}}_{1}^{j,k}$ are successfully fixed, the right ionosphere free combined ambiguity $\nabla {\hat{n}}_{IF}^{j,k}$ of PPP can be obtained:

(5) $$\lambda_{IF}\cdot \nabla {\hat{n}}_{IF}^{j,k}=\frac{cf_{2}}{f_{1}^{2}-f_{2}^{2}}\nabla {\overset{⌢}{n}}_{WL}^{j,k}+\frac{c}{f_{1}+f_{2}}\nabla {\overset{⌢}{n}}_{1}^{j,k}$$

where $\nabla {\overset{⌢}{n}}_{WL}^{j,k}=\nabla N_{r,WL}^{j,k}-\nabla b_{wl}^{j,k}$, $\nabla {\overset{⌢}{n}}_{1}^{j,k}=\nabla N_{r,1}^{j,k}-\nabla b_{1}^{j,k}$.

In the RTPPP treatment process, considering the influence of UPD accuracy on the fixed ambiguities and the guarantee of the uniformity of the real-time algorithm, we took fixed integer ambiguities as a strong constraint of the virtual observation value guide equation instead of directly putting them into the original equation [20,34]:

(6) $$\left[\begin{array}{c} \underset{n\times 1}{V}\\ \underset{n2\times 1}{V} \end{array}\right]=\left[\begin{array}{c} \underset{n\times (5+n)}{A}\\ \underset{n2\times n}{G} \end{array}\right]\left[\begin{array}{c} \underset{5\times 1}{X}\\ \underset{n\times 1}{N} \end{array}\right]-\left[\begin{array}{c} \underset{n\times 1}{L}\\ \underset{n2\times 1}{L} \end{array}\right],\left[\begin{array}{c} \underset{n\times n}{P}\\ \underset{n2\times n2}{P_{G}} \end{array}\right]$$

where A is the coefficient matrix in the PPP process, X is the matrix of the estimated position parameters’ GNSS receiver clock error and the troposphere wet delay in the PPP process and the dimension is 5, N is the matrix of zero-difference ambiguities in the PPP process and the dimension is the number of observed satellite, $\underset{n\times 1}{V}$ is the residuals matrix of observation data, $\underset{n2\times 1}{V}$ is the residuals matrix of virtual observation data, G is the constraint coefficient matrix for introducing the fixed single-difference ambiguities and $P_{G}$ is the corresponding weight matrix, usually set as $P_{G}(i)=10^{4}$, n is the number of observed satellites, n2 is the number of fixed single-difference ambiguities.

### 2.2. UPD Real-Time Estimation for Satellite Terminal

Based on the real-time ambiguities of PPP estimated from a reference network, State Space Representation (SSR) data streams are adopted for real-time precise orbit clock correction. In this study, the UPD estimation strategy proposed by Ge et al. [16] is applied. The single-difference between two satellites is adopted to eliminate the receiver terminal UPD. According to the consistency of UPD influence on various stations, the UPD is separated according to the data of multiple stations. Melbourne-Wübbena (WM) [27,28] combination observation values are adopted to calculate the float ambiguities of single-difference between satellites $\nabla N_{r,WL}^{j,k}$, and multi-epoch is smoothed to obtain $\nabla {\hat{N}}_{r,WL}^{j,k}$ to weaken the influence of measured noise and multi-path error [35]. The smoothed ambiguity and the corresponding noise are as follows:

(7) $$\begin{array}{l} \nabla {\hat{N}}_{r,WL}^{j,k}(i)=\nabla {\hat{N}}_{r,WL}^{j,k}(i-1)+\frac{1}{i}(\nabla N_{r,WL}^{j,k}(i)-\nabla {\hat{N}}_{r,WL}^{j,k}(i-1))\\ \sigma_{i}^{2}=\sigma_{i-1}^{2}+\frac{1}{i}[{\left(\nabla N_{r,WL}^{j,k}(i)-\nabla {\hat{N}}_{r,WL}^{j,k}(i-1)\right)}^{2}-\sigma_{i-1}^{2}] \end{array}$$

where $\sigma_{i}^{2}$ is the error of the i-th epoch. During the smoothing process, the quality of float ambiguities should be controlled to eliminate cycle slip or gross error properly. After the qualified floating wide-lane ambiguity is obtained, the relationship between the UPD and the ambiguity is as follows:

(8) $$\nabla {\hat{N}}_{r,WL}^{j,k}=\nabla {\hat{n}}_{r,WL}^{j,k}+\nabla b_{wl}^{j,k}$$

where $\nabla {\hat{n}}_{r,WL}^{j,k}$ is the most approximate integer of $\nabla {\hat{N}}_{r,WL}^{j,k}$ and can be calculated according to $\nabla {\hat{n}}_{r,WL}^{j,k}=\{\begin{array}{c} \mathrm{int}(\nabla {\hat{N}}_{r,WL}^{j,k}+0.5),\nabla {\hat{N}}_{r,WL}^{j,k}\geq 0\\ \mathrm{int}(\nabla {\hat{N}}_{r,WL}^{j,k}-0.5),\nabla {\hat{N}}_{r,WL}^{j,k}<0 \end{array}$ (int(·) is the integer operator) The value range of $\nabla b_{wl}^{j,k}$ is [−0.5,0.5], and considering that the UPD characteristic that $\nabla b_{wl}^{j,k}\;=\;0.5$ is equivalent to $\nabla b_{wl}^{j,k}\;=\;-0.5$, the sine and cosine function method [24] irrelevant to the integer item should be adopted to estimate $\nabla b_{wl}^{j,k}$:

(9) ∇bwlj,l=arctan{∑s=1s=Numj,kPs⋅sin(2π∇N^r,wlj,k)∑s=1s=Numj,kPs⋅cos(2π∇N^r,wlj,k)}2π

where $Num^{i,j}$ is the number of the stations participating in the calculation and $P_{s}$ is the weight of the decimal part of each measuring station; After $\nabla b_{wl}^{j,k}$ is estimated , $\nabla b_{wl}^{j,k}$ is substituted into Equation (4) to obtain narrow-lane floating-point ambiguity:

(10) $$\nabla {\hat{N}}_{r,1}^{j,k}=\nabla {\hat{n}}_{r,1}^{j,k}+\nabla b_{1}^{j,k}=\frac{f_{1}+f}{f_{1}}\nabla {\hat{N}}_{r,IF}^{j,k}-\frac{f_{2}}{f_{1}^{}-f_{2}^{}}(\nabla {\hat{N}}_{r,WL}^{j,k}-\nabla b_{wl}^{j,k})$$

where $\nabla {\hat{N}}_{r,IF}^{j,k}$ is the float ambiguity of the observation value of the ionosphere free combined model during PPP resolving process, $\nabla {\hat{N}}_{r,1}^{j,k}$ is the single-difference narrow-lane float ambiguity between two satellites, $\nabla {\hat{n}}_{r,1}^{j,k}$ is the most approximate integer of $\nabla {\hat{N}}_{r,1}^{j,k}$, $\nabla b_{1}^{j,k}$ is the single-difference narrow-lane UPD, with the value range of [−0.5,0.5], and $\nabla b_{1}^{j,k}$ is also estimated according to the sine and cosine function method:

(11) $$\nabla {\hat{N}}_{r}^{j,k}+\varepsilon_{r}^{j,k}=\nabla {\hat{n}}_{r}^{j,k}+\nabla b^{j,k}$$

where $\nabla {\hat{N}}_{r}^{j,k}$ is the float ambiguity, $\nabla {\hat{n}}_{r}^{j,k}$ is the most approximate integer of $\nabla {\hat{N}}_{r}^{j,k}$; $\varepsilon_{r}^{j,k}$ is the residual of un-model atmosphere real-time precise orbit clock correction, and they are different from each other in different regions, so the $\nabla b^{j,k}$ could absorb different characteristics of the regional stations is as follow:

(12) $$\nabla b^{j,k}=(\nabla {\hat{N}}_{r}^{j,k}-\nabla {\hat{n}}_{r}^{j,k})+\varepsilon_{r}^{j,k}$$

The UPD estimated by the above method is relative to the reference satellite. For the unification of the UPD datum, the reference satellite UPD should be set to zero as the datum to convert the single-difference UPD $\nabla b_{wl}^{j,k}$ and $\nabla b_{1}^{j,k}$ into zero-difference UPD $b_{wl}^{j}$ and $b_{1}^{j}$. The zero-difference UPDs of all satellites have a parameter bias $b_{wl}^{k}$ and $b_{1}^{k}$, and such bias will be absorbed by the receiver clock correction at the user terminal during positioning. When the reference satellite selected for UPD estimation has been changed, the corresponding bias will be also changed, but it will not influence user positioning. Therefore, there is no need for UPD users to consider the inconsistency of the reference satellite during positioning.

A certain quantity of stations should be selected for UPD estimation, and if more stations are more uniformly distributed around the world, it is more favorable for UPD estimation, and the global UPD estimation model is the same as the regional UPD estimation model, but due to the adoption of single-difference between satellites in the UPD estimation model, globally distributed sites cannot have the same reference satellite, so it is necessary to consider the reference satellite recursive method in the global UPD estimation. Actually, it is difficult to obtain the real-time data of the global stations for UPD estimation, so only regional stations are needed for UPD estimation only serving for the designated regions, and the different distributions of the regional stations can cause different UPD results, thus influence the PPP-AR resolving effect of the reference station.

## 3. Experiment Results and Discussion

**Figure 1:**
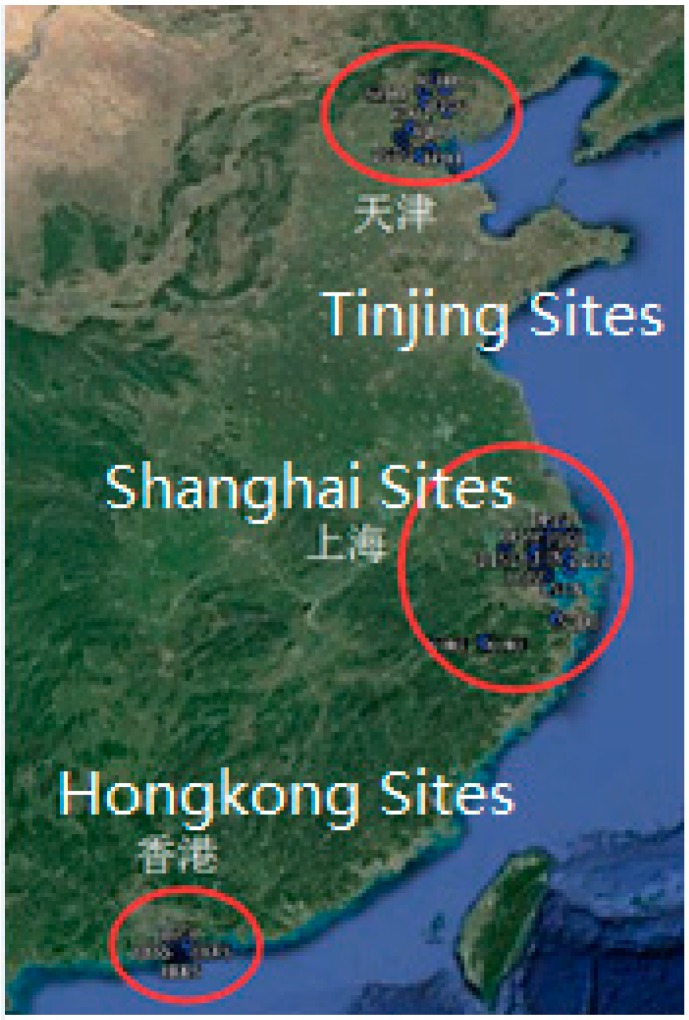
Regional Station Distribution Diagram.

**Figure 2:**
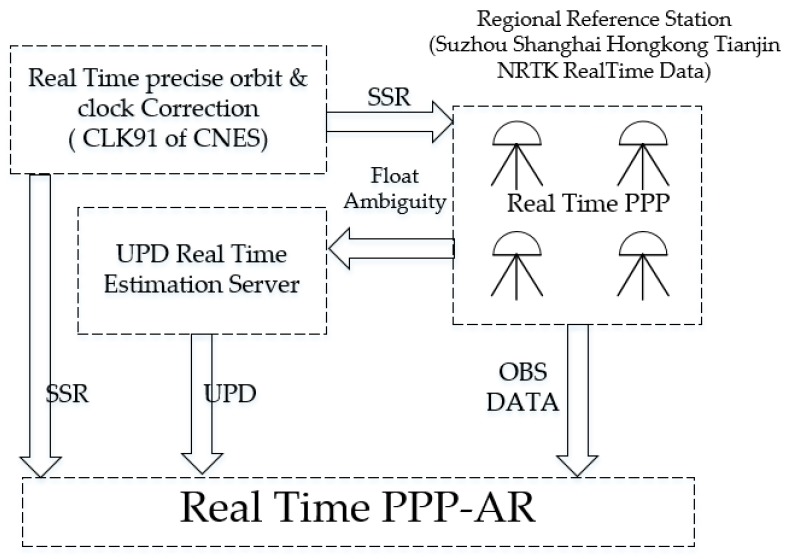
Real-time Analysis Topology.

In order to analyze the properties of the real-time UPD products and their influence on RTPPP-AR, observation data from different regional reference network stations located in Shanghai, Suzhou, Tianjin, Hong Kong, etc. were adopted for the UPD real-time estimation of Global Positioning System (GPS) satellites and RTPPP-AR according to different strategies, wherein the station distribution is as shown in Figure 1. The CLK91 product issued by the Centre National d’Études Spatiales (CNES) was adopted for the real-time satellite precise orbit and clock in the experiment. The real-time analysis topology is as shown in Figure 2.

### 3.1. Real-Time Uncorrected Phase Delay UPD Characteristic of Satellite Terminal

**Figure 3:**
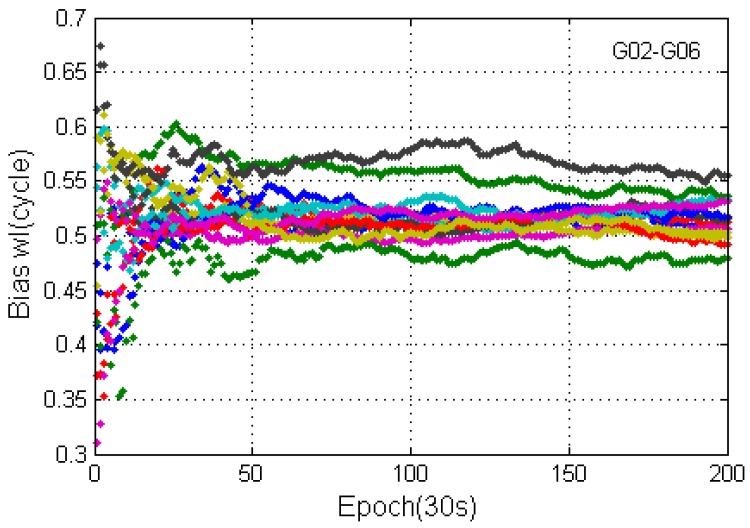
The fractional values of the wide lane ambiguity of G06 (reference satellite G02) at different stations.

**Figure 4:**
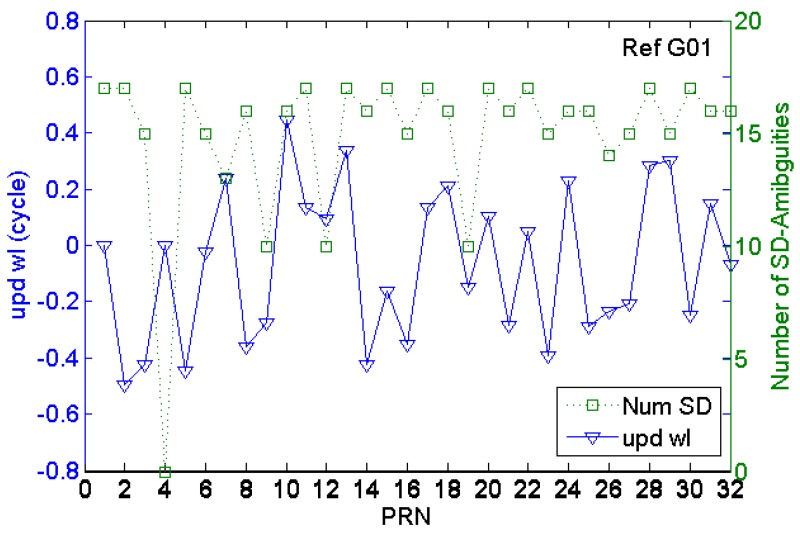
GPS satellite wide-lane ambiguity and number of single-difference ambiguities for calculation.

Figure 3 shows the fractional values of the wide lane ambiguities of the same GPS satellite at different stations, where the consistency can be seen. Figure 4 shows the real-time UPD of each GPS satellite, estimated on the 187th day of 2016 according to 16 reference station networks in Suzhou and Shanghai. As shown in the figure, about 15 effective single-difference ambiguities that pass the data quality control (the quantity of the effective reference stations for the resolving process) participated in the UPD resolving process, thus indicating that the float ambiguities of each satellites in the reference station network were sufficiently adopted, and the decimal part of the wide-lane ambiguities have strong consistency. The wide-lane UPDs of each satellite are in the range of 0.2 cycle~0.5 cycle, and if the UPDs are not corrected in PPP, the wide-lane ambiguity resolution would be significantly influenced. Therefore, before RTPPP-AR calculation, it is necessary to correct the float wide-lane ambiguity by UPD to recover the integer characteristics of ambiguities.

**Figure 5:**
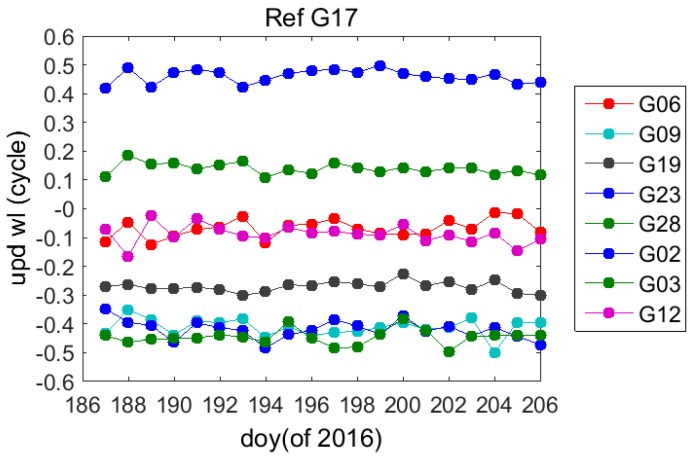
Wide-lane uncalibrated phase delay of the GPS satellite between the 187th and the 206th day of 2016.

The data of the reference stations for 20 days from the 187th day to the 206th day of 2016 were adopted for the real-time wide-lane UPD estimation of the GPS satellite, wherein the statistical results of the wide-lane decimals for multiple days are as shown in Figure 5, where the wide-lane UPD change rate of satellites is less than 0.1 cycle/day. Table 1 shows that standard deviation (STD) of the wide-lane UPD of each satellite for multiple days is less than 0.05 cycle, which indicates a high stability.

**Figure 6:**
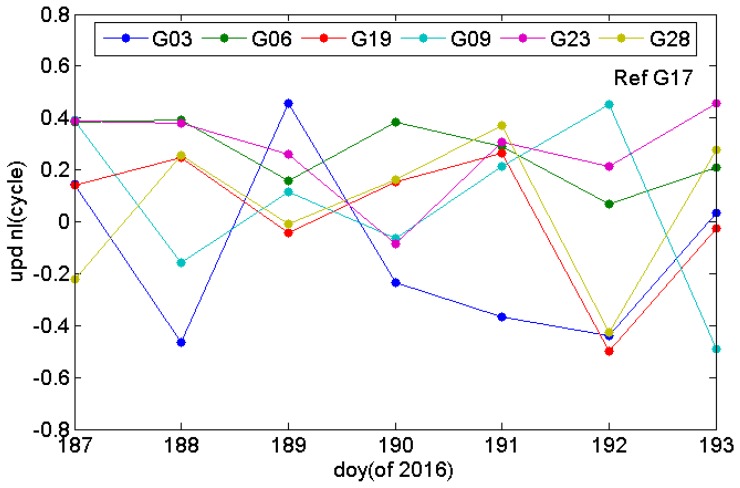
Narrow-lane uncalibrated phase delay of the GPS satellite between the 187th and the 193th day of 2016.

After obtaining the wide-lane UPD, the float ambiguities of the ionosphere-free combined model were adopted to estimate the narrow-lane UPDs of the satellite terminal. Figure 6 shows the narrow-lane UPDs of GPS satellites around 9:00 (GPS time) of each day between the 187th day and the 193th day of 2016. Table 2 shows that standard deviation (STD) of the narrow-lane UPD of each satellite for multiple days. According to Figure 6 and Table 2, the narrow-lane UPDs are inferior to the wide-lane UPD in daily stability and have significant change. The wavelength of GPS narrow-lane is about 10.6 cm, due to the short wavelength, narrow-lane UPDs are easy to be affected by the ionosphere.

### 3.2. UPD Estimated by Regional Stations for RTPPP-AR Positioning Experiments

For RTPPP-AR positioning, firstly it is necessary to obtain the UPD of the satellite estimated by the reference network stations in a real-time manner to recover the integer characteristics of the zero-difference ambiguities, but the different distributions of the reference network stations used for the real-time UPD estimation will cause different UPD results, thus influencing the RTPPP-AR resolving effect of the reference stations.

About 37 CORS stations in Tianjin, Shanghai, Hong Kong, etc. were selected for analysis as follows, and this experiment is based on the Precise Point Positioning based on the RTK networks (PPP-RTK) system developed independently by ourselves, so the reference site coordinates are known. Therefore, the reference station coordinates are set to known values in the UPD estimation and RTPPP-AR processing.

Firstly, UPDs estimated by the reference stations were divided into three models to analyze the influence of the real-time UPD estimation of different regional stations on RTPPP-AR positioning. Specifically, relevant information of the three models is as shown in Table 3.

**Figure 7:**
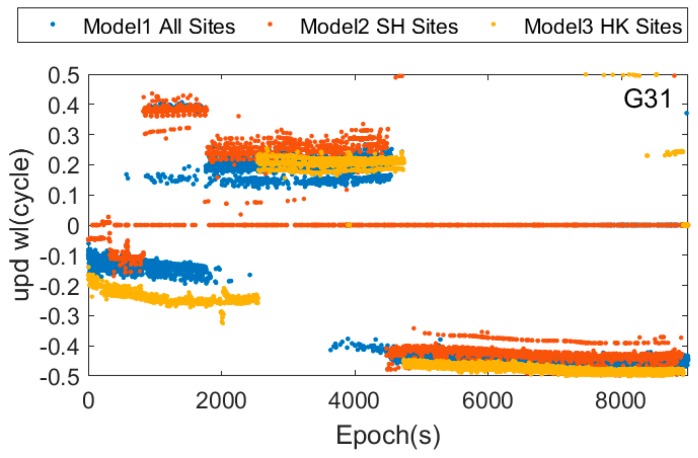
Wide-lane uncorrected phase delay of G31 in three UPD models.

**Figure 8:**
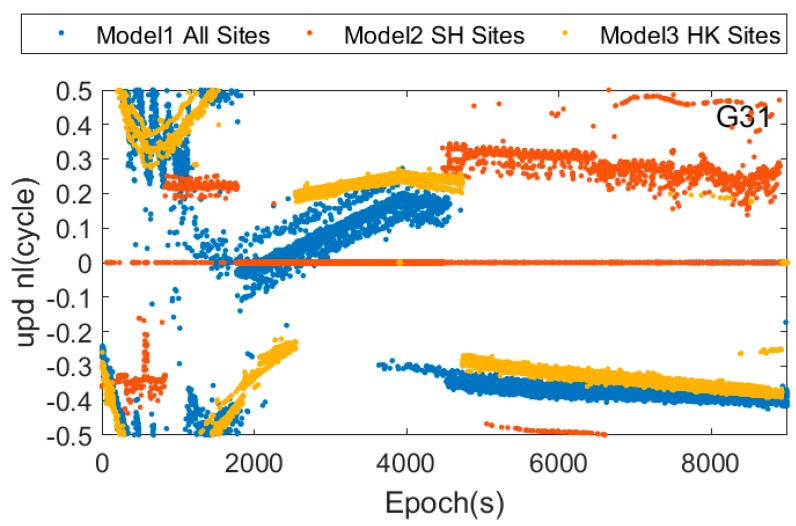
Narrow-lane uncorrected phase delay of G31 in three UPD models.

Figure 7 and Figure 8 show the real-time estimated UPD results of the G31 satellite in the different UPD modes, and which shows that the UPD results in this three modes are not the same. This difference is mainly caused by the different atmospheric environment in different regions sites, and the un-model ionosphere and troposphere delay are absorbed by UPD when estimating UPDs.

The epoch ambiguity fixed rates of RTPPP-AR positioning results under above the three models are as shown in Table 4. If the ambiguities are fixed of one epoch, we think the epoch has been fixed, so the epoch ambiguity fixed rates is shown as follows:

(13) $$rate=\frac{\sum Fixed\_Epoch}{\sum Epoch}\;\times \;100\%$$

**Figure 9:**
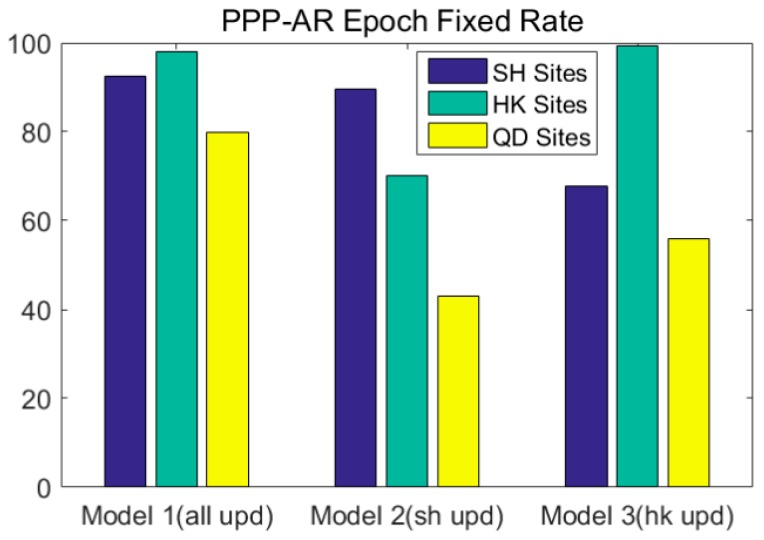
RTPPP-AR average percentage ambiguity fixed rates of different regional stations in different UPD models (reference station coordinates are set to known).

According to Table 4, for the UPD estimation and RTPPP-AR calculation achieved by all stations in Model 1, the average fixed rates of the stations in Shanghai, Hong Kong and Tianjin are respectively 92.5%, 97.9% and 79.9%; for the UPD estimation and RTPPP-AR calculation achieved by the stations in Shanghai in Model 2, the average fixed rates of the stations in Shanghai, Hong Kong and Tianjin are 89.5%, 70.2% and 42.9% respectively; For the UPD estimation and RTPPP-AR calculation achieved by the stations in Hong Kong in Model 3, the average fixed rates of the stations in Shanghai, Hong Kong and Tianjin are respectively 67.7%, 99.3% and 55.8%, as shown in Figure 9. Therefore, the different UPD models causes significantly influence RTPPP-AR fixed rate.

**Figure 10:**
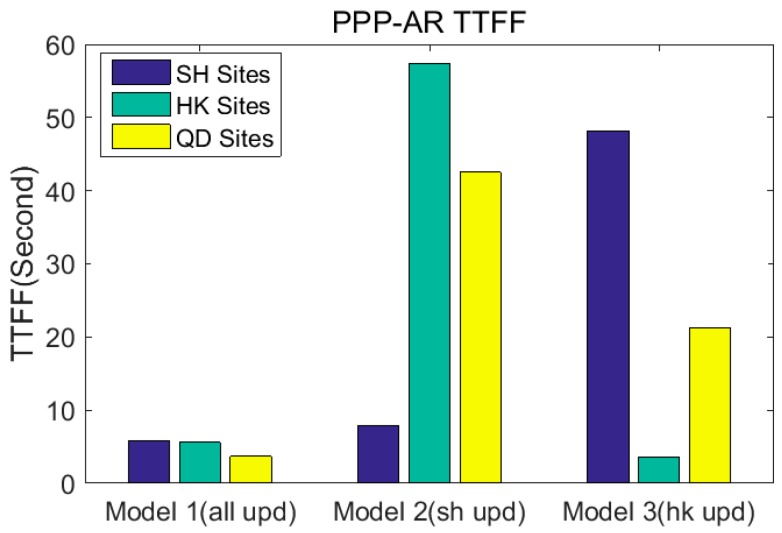
Average TTFF for RTPPP-AR of different regional stations in different UPD models (reference station coordinates are set to known).

According to the Table 5, the average Time to First Fix (TTFF) of the stations in Shanghai, Hong Kong and Tianjin are 5.8 s, 5.6 s, 3.6 s respectively in Model 1; the average TTFF of the stations in Shanghai, Hong Kong and Tianjin are 7.9 s, 57.3 s, 42.5 s respectively in Model 2; the average TTFF of the stations in Shanghai, Hong Kong and Tianjin are respectively 48.2 s, 3.6 s, 21.2 s in Model 3, as shown in Figure 10.

According to Table 4 and Table 5, Figure 9 and Figure 10, the RTPPP-AR resolving performance of the reference stations is obviously related to the stations used for UPD estimation. The real-time UPD products employing the stations in certain regions for estimation have a poor fixed rate for the RTPPP-AR ambiguities of other regional stations and the TTFF is longer, but has a good fixed rate for the RTPPP-AR ambiguities of the stations in the same region and the TTFF is shorter. Therefore, for RTPPP-AR calculation in a large scope, the sparse stations in the user region should be provided for the calculation of the real-time UPD product to improve the RTPPP-AR resolving performance of the user region. In other words, the availability of such derivative services as PPP based on RTK networks (PPP-RTK) [36,37,38] regional augmentation system provided by RTPPP-AR could be influenced. Some PPP-RTK systems run the zero-difference RTPPP-AR in the stations to fix the zero-difference ambiguities, then obtain the zero-difference augmentation information of observation data. The users log into the system to get the zero-difference augmentation information for zero-difference positioning like PPP. In light of the above, the improvement of the ambiguity fixed rate and TTFF is helpful to the application of PPP-RTK using the fixed ambiguities information of PPP.

Theoretically, the UPD of the satellite is independent of the regions, and UPD of each satellite should have uniformity for the global users, but due to the influence of the real-time precise orbit clock correction adopted and the station distribution, UPD estimation has certain regional characteristics. Moreover, the atmosphere delays in different regions are also different from each other, so the estimated UPD could absorb different characteristics of the regional stations. Therefore, for the RTPPP-AR calculation in a large scope for relevant service, it is necessary to sparsely set reference stations in the service regions for the UPD calculation in a large scope to fit the difference brought by different regional stations to improve the RTPPP-AR fixed rate and TTFF of the stations in a large scope.

**Figure 11:**
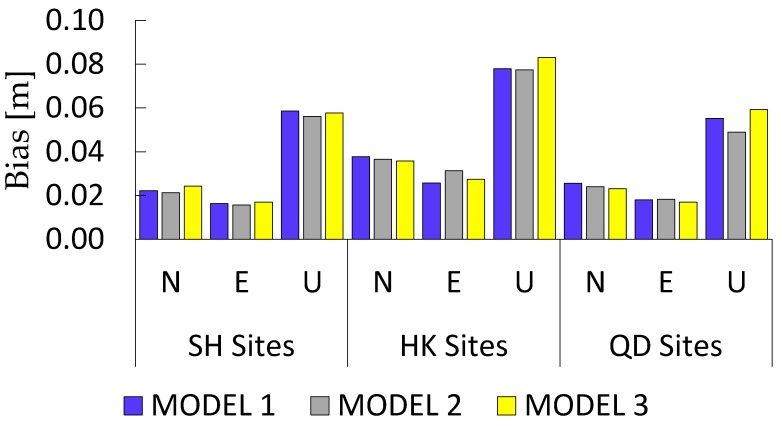
RTPPP-AR positioning accuracies of different regional stations in different models.

RTPPP-AR resolution positioning accuracies in different models are shown in Table 6 and Figure 11. The positioning accuracies using different UPD models are the root mean square (RMS) of the positioning results of the ambiguities-fixed epochs. Obviously, the dynamic resolution accuracies in different UPD models are basically equivalent, thus indicating that the accuracies in different models are not essentially different from each other after the ambiguities are correctly fixed.

## 4. Conclusions

In this paper, RTPPP-AR was theoretically analyzed and a method for estimating the UPD in a real-time manner from network PPP solutions was also explained; the data of the regional reference stations were adopted to estimate UPD in order to analyze the real-time UPD product property. Additionally, UPD estimations of different regional stations were adopted for RTPPP-AR positioning to obtain the following conclusions:

Wide-lane UPD real-time products have a daily change rate of less than 0.1 cycle and STD less than 0.05 cycle, thus presenting high stability; the narrow-lane UPD real-time products show significant change in a day, but have short-term stability, so the period-estimation is suggested for narrow-lane UPD real-time products.

When the stations in the user terminal region participate in the resolution process of the UPD real-time products, the RTPPP-AR fixed rate and TTFF can be significantly improved, thus indicating that the regional UPD real-time products have obvious spatial properties. Therefore, for the RTPPP-AR calculation in a large scope for relevant service, such as PPP-RTK, it is necessary to sparsely set reference stations in the service regions for the UPD estimation in a large scope to fit the difference brought by different regional stations to improve the RTPPP-AR ambiguities fixed rate and TTFF of the stations in a large scope.

The dynamic RTPPP-AR accuracies of the regional stations in different UPD models are basically equivalent, which shows that the accuracies in different UPD models are not essentially different from each other after the ambiguities is correctly fixed, namely, such dynamic accuracy is better than 3 cm in the plane and 8 cm in the upward direction.

## Figures and Tables

**Table 1:** Wide-lane uncalibrated phase delay STD of the GPS satellite between the 187th and the 206th day of 2016.

PRN	G02	G03	G06	G09	G12	G19	G23	G28
STD [cycles]	0.03	0.03	0.03	0.03	0.03	0.02	0.02	0.02
STD [m]	0.026	0.026	0.026	0.026	0.026	0.017	0.017	0.017

**Table 2:** Narrow-lane uncalibrated phase delay STD of the GPS satellite between the 187th and the 193th day of 2016.

PRN	G03	G06	G09	G19	G23	G28
STD [cycles]	0.32	0.12	0.31	0.24	0.17	0.27
STD [m]	0.034	0.013	0.032	0.026	0.018	0.029

**Table 3:** Calculation Strategy Information Sheet for Different UPD Estimation Models.

Model	Stations for UPD Estimation	Strategy Information
Model 1: ALL Sites UPD Model	37 stations in total	All 37 stations were adopted for UPD estimation, and the UPD results were adopted for RTPPP-AR calculation of all stations.
Model 2: Shanghai UPD Model	YIWU, XUCH, CJZZ, DING, DPZZ, FUQI, JD01, JSWZ, JZZZ, LJIN, LNGN, XTZZ, ZQZZ (14 stations)	14 stations in Shanghai regions were adopted for UPD estimation, and the UPD results were adopted for RTPPP-AR calculation of all stations.
Model 3: Hong Kong UPD Model	HKKS, HKKT, HKLM, HKMW, HKNP, HKOH, HKPC, HKSC, HKSL, HKSS, HKST, HKTK, HKWS (13 stations)	13 stations in Hong Kong were adopted for UPD estimation, and the UPD results were adopted for RTPPP-AR calculation of all stations.

**Table 4:** Percentage of epoch ambiguity fixed rates for RTPPP-AR of Different Regional Stations in Different UPD Models (reference station coordinates are set to known).

Station		Model		Station		Model		Station		Model	
	1	2	3		1	2	3		1	2	3
YIWU	95.6	90.3	80.9	HKKS	92.3	69.2	98.9	SZ01	85.8	52.8	52.5
XUCH	87.8	75.8	59.1	HKKT	98.8	74.0	99.0	TG01	88.0	46.1	57.6
CJZZ	91.3	93.3	74.2	HKLM	99.1	83.3	99.8	TGT0	80.7	57.0	56.8
DING	89.9	84.6	55.9	HKMW	91.0	63.3	98.8	TP01	86.6	68.8	56.0
DPZZ	96.0	96.4	75.9	HKNP	99.2	78.0	99.2	WK01	73.6	46.7	47.5
FUQI	77.1	86.2	23.3	HKOH	98.7	64.1	99.0	XLH0	86.5	62.8	51.4
JD01	92.8	92.3	56.9	HKPC	99.3	70.3	99.6	XQ01	61.1	39.7	35.7
JSWZ	92.8	93.4	76.3	HKSC	99.3	73.7	99.8	XY01	83.4	65.9	76.2
JZZZ	96.2	93.3	88.3	HKSL	99.2	56.8	99.8	YL01	81.7	69.3	57.5
LJIN	96.8	92.8	49.0	HKSS	99.1	65.0	99.3	BD01	60.7	28.6	28.4
XTZZ	96.3	92.7	93.7	HKST	99.0	69.8	99.4	DG01	90.4	66.5	50.6
ZQZZ	97.0	82.6	79.2	HKTK	98.0	72.9	99.1				
				HKWS	99.4	71.5	99.4				
Average	92.5	89.48	67.7	Average	97.9	70.2	99.3	Average	79.9	42.9	55.8
Stations in Shanghai, etc. (SH Sites)				Stations in Hong Kong (HK Sites)				Stations in Tianjin (QD Sites)			

**Table 5:** TTFF for RTPPP-AR of different regional stations in different UPD models (reference station coordinates are set to known).

Station		Model		Station		Model		Station		Model	
	1	2	3		1	2	3		1	2	3
YIWU	26	1	79	HKKS	1	92	1	SZ01	1	42	37
XUCH	2	14	89	HKKT	1	12	2	TG01	3	47	68
CJZZ	17	15	51	HKLM	3	50	2	TGT0	4	45	7
DING	2	1	54	HKMW	13	84	11	TP01	1	63	5
DPZZ	1	9	33	HKNP	24	83	2	WK01	17	26	24
FUQI	7	5	29	HKOH	3	88	8	XLH0	9	52	24
JD01	6	23	82	HKPC	5	84	3	XQ01	1	49	3
JSWZ	2	7	31	HKSC	1	15	1	XY01	1	26	10
JZZZ	2	3	36	HKSL	2	75	1	YL01	1	46	11
LJIN	1	8	34	HKSS	1	82	1	BD01	2	21	26
XTZZ	2	5	35	HKST	12	23	10	DG01	1	50	18
ZQZZ	1	4	25	HKTK	1	86	1				
				HKWS	1	6	1				
Average	5.8	7.9	48.2	Average	5.6	57.3	3.6	Average	3.7	42.5	21.2
Stations in Shanghai, etc. (SH Sites)				Stations in Hong Kong (HK Sites)				Stations in Tianjin (QD Sites)			

**Table 6:** Statistical sheet for RTPPP-AR positioning accuracies of different regional stations in different models.

Regional Station	Bias [m]	MODEL 1	MODEL 2	MODEL 3	Average [m]
SH Sites	N	0.022	0.021	0.024	0.022
	E	0.016	0.016	0.017	0.016
	U	0.059	0.056	0.058	0.058
HK Sites	N	0.038	0.037	0.036	0.037
	E	0.026	0.031	0.027	0.028
	U	0.078	0.077	0.083	0.079
QD Sites	N	0.026	0.024	0.023	0.024
	E	0.018	0.018	0.017	0.018
	U	0.055	0.049	0.059	0.054
